# INFERENCE: An Evidence-Based Approach for Medicolegal Causal Analyses

**DOI:** 10.3390/ijerph17228353

**Published:** 2020-11-11

**Authors:** Putri Dianita Ika Meilia, Maurice P. Zeegers, Herkutanto, Michael Freeman

**Affiliations:** 1 Care and Public Health Research Institute (CAPHRI), Faculty of Health, Medicine and Life Sciences, Maastricht University Medical Center+, Universiteitssingel 40, 6229 ER Maastricht, The Netherlands; m.zeegers@maastrichtuniversity.nl (M.P.Z.); m.freeman@maastrichtuniversity.nl (M.F.); 2 Department of Forensic Medicine and Medicolegal Studies, Faculty of Medicine, University of Indonesia, Jl. Salemba Raya No. 4, Salemba, Jakarta Pusat 10430, Indonesia; herkutanto@yahoo.co.id

**Keywords:** forensic medicine, medicolegal analysis, causal analysis, comparative risk, evidence-based practice

## Abstract

A fundamental purpose of forensic medical, or medicolegal, analysis is to provide legal factfinders with an opinion regarding the causal relationship between an alleged unlawful or negligent action and a medically observed adverse outcome, which is needed to establish legal liability. At present, there are no universally established standards for medicolegal causal analysis, although several different approaches to causation exist, with varying strengths and weaknesses and degrees of practical utility. These approaches can be categorized as intuitive or probabilistic, which are distributed along a spectrum of increasing case complexity. This paper proposes a systematic approach to evidence-based assessment of causation in forensic medicine, called the INtegration of Forensic Epidemiology and the Rigorous EvaluatioN of Causation Elements (INFERENCE) approach. The INFERENCE approach is an evolution of existing causal analysis methods and consists of a stepwise method of increasing complexity. We aimed to develop a probabilistic causal analysis approach that (1) fits the needs of legal factfinders who require an estimate of the probability of causation, and (2) is still sufficiently straightforward to be applied in real-world forensic medical practice. As the INFERENCE approach is most relevant in complex cases, we also propose a process for selecting the most appropriate causal analysis method for any given case. The goal of this approach is to improve the reproducibility and transparency of causal analyses, which will promote evidence-based practice and quality assurance in forensic medicine, resulting in expert opinions that are reliable and objective in legal proceedings.

## 1. Introduction

Forensic medicine is a branch of medicine that applies medical knowledge and technology to aid in legal matters [1,2]. Although the scope and role of forensic medicine practices are highly diverse [3], a common fundamental purpose of forensic medical, or medicolegal, analysis is to provide legal factfinders with an opinion regarding the causal relationship between an alleged unlawful or negligent action and a medically observed adverse outcome (injury, disease, or death). Forensic medical expert opinions regarding causation are a crucial element in legal proceedings, as they explain and quantify the relationship between a potentially harmful act and an adverse outcome, which is needed to establish legal liability [4]. Causation differs from diagnosis in that it cannot be directly observed. It is usually established by comparing the risk of injury from a harmful event versus the risk of the same injury at the same point in time but in the absence of the harmful event, given what is known about the individual’s condition and circumstances. This approach is called “counterfactual causation” [5,6,7,8] because it does not merely ask “*what is the chance of injury from the adverse event?*” but also incorporates the hypothetical question of “*what was the chance of injury if the adverse event had not occurred*?” While causation of individual injury, disease, and death is the focus of forensic medical analysis (specific causation), the cause of disease and injury in populations (general causation) is also investigated, using epidemiologic methods and data.

At present, there are no universally established standards for medicolegal causal analysis, although several different approaches to causation have been described in the literature with their strengths and weaknesses and varying degrees of practical utility [9]. In cases of increased causal complexity, forensic medical practitioners face a choice as to which approach is the most practical yet robust. Therefore, a systematic approach is needed to (1) choose the most appropriate method of causal analysis for a case and (2) properly apply the chosen method to determine causality and aid legal decision making.

In the present discussion, we propose a systematic approach to evidence-based assessment of causation in forensic medicine, called the INtegration of Forensic Epidemiology and the Rigorous EvaluatioN of Causation Elements (INFERENCE) approach. As the INFERENCE approach is most relevant in complex cases, we also propose a process for selecting the most appropriate causal analysis method for any given case. The goal of the INFERENCE approach is to improve the reproducibility and transparency of causal analyses, which will promote evidence-based practice and quality assurance in forensic medicine, resulting in expert opinions that are reliable and objective in legal proceedings.

## 2. Current Methods of Medicolegal Causal Analysis

There are two broad categories of causation: specific/individual and general. In some circumstances, there is a little overlap between specific and general causation, e.g., the cause of the death of a person who has a knife wound to the chest that has pierced the left ventricle has no readily apparent epidemiologic features. On the other hand, the death of a lifetime smoker from lung cancer can only be said to be caused by smoking based on what is known from epidemiologic studies, which have shown that the risk of developing lung cancer is 25 times higher in smokers than nonsmokers [10]. In such a situation, what we know about the cause of disease in populations translates to what we can infer about the cause of an individual’s disease because we treat the individual similar to a randomly selected member of the population. Thus, the epidemiologic evidence that any lifetime smoker, similar to the individual, is 25 times more likely to get lung cancer serves as reliable (and legally admissible) evidence that the lung cancer in the individual is 25 times more likely caused by his smoking, versus any other cause [11]. This translatability from general causation (“can it?” and “does it?”) to specific causation (“did it?”) is the most common situation found in legal proceedings.

An essential element of all causation determinations is the comparison of risks, even if this fact is not explicitly mentioned. In legal terms, this comparison of risks usually takes the form of determining liability based on the balance of probabilities (in civil litigations) or guilt beyond reasonable doubt (in criminal proceedings). As an illustration, even the knife wound example above is one that involves at least a mental acknowledgment of risk disparity. The fact that the ventricle was pierced allows for the inference that there is a nearly 100% risk of near-instantaneous death from such a wound, and in the absence of a similar degree of risk from an alternative cause of death occurring at the same time (e.g., a gunshot wound to the head), no further consideration of other competing causes of the death is warranted. If the knife wound had not pierced the ventricle, however, we would be less sure about the most likely cause of death, especially if there were other competing causes of death present as well (e.g., if the decedent had fallen a distance before being stabbed and was found to have intracranial hemorrhage at autopsy). Whether he would have died from the fall in the absence of the knife wound is a question that can only be evaluated by quantifying and comparing the risk of the two events.

Depending on the nature of the analysis and underlying facts, the degree to which a medicolegal assessment of causation requires the application of epidemiologic concepts and data directed at the quantification and comparison of risk ranges from none to very high. The discipline that is concerned with the use of epidemiology within the practices of forensic medicine is forensic epidemiology (FE). FE has been described as a systematic approach to incorporating population-based methods, principles, and data in the process of medicolegal causal evaluation [12,13,14,15,16,17]. An FE analysis commonly aims at quantifying a counterfactual causal probability, appropriate for presentation in a civil or criminal legal proceeding [16]. This counterfactual causal probability is presented in the form of a comparative risk ratio (CRR), which is the quantification of the probability of the adverse outcome of interest (p[O]) given the harmful exposure (|E) (as the numerator) versus the probability of the adverse outcome in the absence of the harmful exposure (|$\bar{E}$) (as the denominator) [15]. In mathematical terms, the CRR can thus be expressed as the following equation:

$$\mathrm{CRR}=\;\frac{p\left(O|E\right)}{p\left(O|\;\bar{E}\right)}\;=\;\frac{\mathrm{The}\;\mathrm{probability}\;\mathrm{of}\;\mathrm{the}\;\mathrm{injury}\;\mathrm{outcome}\;\mathrm{given}\;\mathrm{the}\;\mathrm{exposure}}{\mathrm{The}\;\mathrm{probability}\;\mathrm{of}\;\mathrm{the}\;\mathrm{injury}\;\mathrm{outcome}\;\mathrm{given}\;\mathrm{no}\;\mathrm{exposure}\;\mathrm{or}\;\mathrm{competing}\;\mathrm{causes}\;}$$

In a review of existing approaches to medicolegal causal analysis [9], we found that previously described causal analysis methods in forensic medicine can be categorized as intuitive or probabilistic and that the array of causal analysis methods are distributed along a spectrum of increasing complexity. The table in Appendix A summarizes current methods of medicolegal causal analysis. Purely intuitive methods generally do not include explicit efforts to quantify the degree of a causal association. Additionally, intuitive methods do not typically follow a systematic approach, including the application of the Bradford-Hill viewpoints (also known as the “Hill criteria”) [18], which are used to assist in the evaluation of the plausibility and strength of investigated associations. In contrast with purely intuitive methods, probabilistic methods typically require a higher degree of epidemiologic or other scientific evidence, as opposed to relying solely on common sense combined with individual experience and training. Probabilistic causal approaches are more likely to result in a causal probability, and more likely to require the application of the Hill criteria. Systematic approaches to causation are never purely intuitive but may involve a hybrid between intuitive and purely probabilistic methods. The INFERENCE approach described in the following discussion is a systematic approach that is used when quantification of a causal probability is required for the specific circumstances of an investigation. Table 1 provides a comparison of the different elements required for the spectrum of categories of causal analysis methods.

## 3. The INtegration of Forensic Epidemiology and the Rigorous EvaluatioN of Causation Elements (INFERENCE) Approach and the Approach Selection Procedure

Based on the results of our prior review [9], we have combined the most useful elements of existing methods to form a practicable probabilistic approach for causal analysis in complex cases, i.e., the INFERENCE approach, to which the remainder of this paper is devoted. The INFERENCE approach is an evolution of existing causal analysis methods, including Forcier–Lacerte’s analysis of causation elements [19], Hill’s viewpoints [6,18,20], and basic principles of FE in evaluating individual causation [13,15,16,17,21]. Our goal was to develop a probabilistic causal analysis approach that (1) fits the needs of legal factfinders who require an estimate of the probability of causation in order to make ultimate determinations of liability, negligence, and damages in civil matters, as well as guilt or innocence in criminal matters, and (2) is sufficiently straightforward to be applied in real-world forensic medical practice. To that end, we have avoided an approach that is technically too complicated to be applied and explained by the forensic medical practitioner with a basic level of epidemiological and statistical knowledge (as can be expected based on his/her medical training). *N.B.*: in the following discussion the term “harmed party” refers to both “plaintiff/claimant” (in civil litigations) and “victim” (in criminal proceedings). “Defendant” describes the defendant in either a civil or criminal matter. The causal analysis consists of a stepwise approach as outlined below. To aid comprehension, we will use an example case throughout the description of each step of the INFERENCE approach. The case is that of an infant suffering from hypoxic-ischemic encephalopathy (HIE) after placental abruption with other present risk factors, including chorioamnionitis, abnormal fetal heart rate, and maternal obesity.

### 3.1. Formulation of the Causal Question to be Investigated

The first step in every causal analysis is to 5,6,7,8formulate the causal question based on the causal theories/hypotheses of the opposing parties. The counterfactual hypotheses may be mutually exclusive, although they may overlap to some degree, and they do not have to be exhaustive [22,23]. A well-defined causal question not only asks, “What is the probability that B was caused by A?” but incorporates counterfactual scenarios, i.e., “How much more probable is it that B was caused by A compared to by other than A?” or “What is the probability that but for A, B would not have occurred?”. The causal question is then used to compare the opposing theories and to assess the need for a particular causal analysis method. Intuitive methods usually stop after this step because the risk of B given A is so much greater than the risk of B given other than A (or had A not occurred), as in our knife-wound example in the previous section. On the other hand, the causal question in the example case is more complex, i.e., “In an infant exposed to chorioamnionitis, abnormal fetal heart rate, and maternal obesity, how much greater is the risk of HIE after placental abruption compared to if there was no placental abruption?”. As shown by the complexity of the causal question, a more analytic method is needed. Hence, we proceed to the next step.

### 3.2. Consideration of Examination Findings, Injury/Pathophysiologic Mechanism, and Predictive Demographics and History

In this step, we assess every fact of the case and consider its contribution to causation, including all examination findings and the relevant injury/pathophysiologic mechanism. For example, in the knife-wound example, the autopsy shows that the knife has indeed penetrated the left ventricle of the heart, accompanied by a fatal level of blood loss. Additionally, the laceration of the ventricle wall is clear-cut and not frayed, so that we can rule out a spontaneous rupture. Neither do we find any other pathologies that could have caused, or contributed to, death. We might also consider predictive demographics (such as sex, age, and ethnic background) and medical/personal history to strengthen the possibility of causation. For example, spontaneous ruptures of the heart chambers are more common in elderlies with a history of myocardial infarction. Therefore, in a young individual without any known morbidities, the knife wound versus spontaneous rupture question can be answered by considering the abovementioned factors. In more complex cases, however, this step might not be sufficient to point to a specific cause, and a probabilistic approach is required. In the HIE example, we find that, based on existing medical records of the mother and the baby, a placental abruption was indeed diagnosed, in addition to chorioamnionitis and abnormal fetal heart rate. Furthermore, maternal obesity is also present as a predictive factor for fetal HIE.

**Figure 1:**
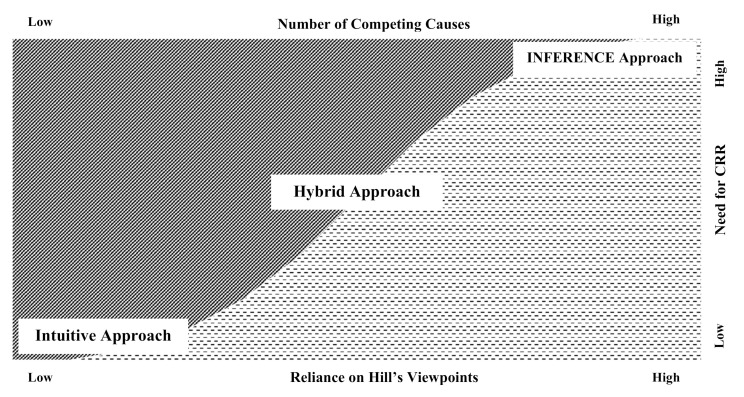
Which causal analysis approach is the most suitable for the specific case?

Before proceeding to the next step, a determination of the necessity for a probabilistic, or even an INFERENCE analysis, is required. Figure 1 depicts the hypothetical distribution of cases according to the following factors:

- The number of competing causes
- The need for an inventory of the Hill causal viewpoints. For a novel or otherwise previously unestablished causal relationship, an analysis of the relevant Hill viewpoints may be required to establish whether the relationship is plausible (please refer to Appendix B for an overview of the Hill viewpoints)
- The need to quantify and compare risks via a CRR approach.

In the HIE case, we have established that there are several known competing causes (placental abruption, chorioamnionitis, abnormal fetal heart rate, and maternal obesity). Furthermore, because the contribution of those factors to HIE, both individually and combined, is not readily apparent, we need to rely on Hill’s viewpoints to establish any causal relationship between them and HIE. We also need to establish whether placental abruption is the *most probable* cause of HIE in this case and to quantify the additional risk of placental abruption in causing HIE compared to when there is no placental abruption. Therefore, because the focus of the analysis is not whether those conditions *can* cause HIE, but rather the *increase* in risk due to placental abruption complicating the other pre-existing conditions, it is appropriate to use a probabilistic method. The next steps are as follows:

### 3.3. Definition of the Medicolegal Causation Elements (MCE)

The MCE is formulated by reconstructing the harmed party’s legal claims regarding causation into a verifiable argument structure. This step consists of defining the following elements [19,22]:

- Definition of the condition of interest (COI):
  The exact nature of the COI at the time it is being assessed should be described. This description includes the anatomical location, pathological features (i.e., fracture, laceration), level of severity, and natural history and sequelae (i.e., spinal cord injury, sudden cardiac death), based on the review of available medical evidence. If an examination of the harmed party is also performed as a part of the analysis, the current condition and functional impairments, therapeutic options, and prognosis may also be described in some cases. The opposing parties should generally agree on this definition of the COI before it can be used in the next steps, although this may not always be possible. The COI might not be the most current condition of the harmed party, but it is the condition that is thought to be caused by the alleged harmful exposure. In the example, the COI is fetal HIE as diagnosed using the appropriate medical criteria.
- Definition of the alleged primary harmful exposure (HE):
  The HE is a description of the alleged cause of the COI by the claiming party, including, when relevant, the nature, level of severity, and timing or temporal association of the defendant’s actions of interest, as well as the mechanism by which the actions caused the alleged harm/injury. This description, too, should be sufficiently well defined and agreed upon by the opposing parties. The HE in the example is placental abruption.
- Definition of potential competing causes (non-HE):
  ○ The pre-exposure health status: as the harmed party’s current health status, relevant to the COI, will be compared to their health status prior to the HE, their pre-existing health status must be described in sufficient detail to allow for a pre-HE/post-HE comparison of the health status. This description should include any pre-existing diseases/impairments/conditions that could have caused or contributed to the COI in the absence of the HE, or which could have interacted with the HE to a substantial degree. Chorioamnionitis, abnormal fetal heart rate, and maternal obesity can all be classified as factors of the pre-exposure health status (pre-existing conditions) in the example case.
  ○ Intervening events: any events that could have contributed to or interacted with the COI, occurring either after the HE and before the first indication of the COI, or after the COI but potentially acting as a modifier of the condition, should be listed. In the example, this could be in the form of a delay in properly treating the placental abruption.

### 3.4. Comparative Risk Assessment of Competing Causes

In this step, we assess the plausibility and temporality of an alleged causal relationship, as follows:

- Plausibility assessment: after the primary HE and all other potential non-HE causes of the COI have been identified, a plausibility assessment must first be performed on the relationship between the COI and the HE, and then on each discrete non-HE cause in order to evaluate the evidence for general causation [19,24,25]. The goal of this part of the analysis is to assess the pathophysiological plausibility of the injury mechanism based on available scientific literature as well as clinical expertise [24] and to avoid a *post hoc ergo propter hoc* fallacy (in which a biologically implausible causal relationship is erroneously deemed to be causal, solely because the effect followed the event in time) [25]. Obviously implausible, trivial, or temporally remote proposed non-HE causes can be eliminated from consideration in this step as well [24]. In the HIE case, all risk factors that were present (i.e., placental abruption, chorioamnionitis, abnormal fetal heart rate, and maternal obesity) can plausibly cause HIE through known pathophysiologic principles, whether individually or in various combinations [26,27,28,29,30,31].
- Temporality assessment: it is essential to establish the timing of the alleged harmful exposure and the first symptom/sign of the injury, for several reasons [24,25]. First, the temporal sequence of the injury following the harm must be correct. Second, the evidence of injury must occur within the effect range of the harmful exposure (“sufficiency”), and the latency between exposure and the first indication of injury must be quantified. The latency is then used to estimate the “hazard-period” between the HE and the first manifestation of the COI [22,25]. From a temporal point-of-view, the risk factors present in the HIE case all precede the occurrence of HIE within a sufficient range of time, as based on medical record data, so temporality can also be established.

During this step, additional analyses from other disciplines may be employed. For example, expertise from clinical specialists about the nature and severity of the COI, information regarding the characteristics of the HE and other competing causes from other relevant disciplines, including pharmacology, toxicology, ballistics, and biomechanics, as well as the plausibility of the COI resulting from the HE and other competing causes.

If the complexity of the case, and the legal process, demands a quantification of the comparative risk, we move on to the core of the INFERENCE approach, i.e., the calculation of the CRR, as follows (note: for ease of comprehension, the detailed calculations used to obtained the values mentioned below are not shown):

- Determination of the CRR numerator value: the numerator is obtained from the risk of the ‎COI given the HE, which is quantified from available epidemiologic data. For the HIE case, ‎epidemiologic data show that the risk of HIE in a neonate exposed to placental abruption in ‎addition to chorioamnionitis, abnormal fetal heart rate, and maternal obesity is 14.9% (1 in ‎‎6.7)‎.
- Determination of the CRR denominator value: the denominator represents either the risk of the condition due to a risk that is attributable to a discrete event (a non-HE) or the pre-COI health status and natural history of the individual (the “base risk”). The latter is estimated from epidemiologic data of the cumulative risk of the COI occurring in the hazard period in association with all other potential causes but in the absence of the HE. The estimation of base risk from epidemiologic data is typically based on the assumption that the base risk is relatively uniform over time, allowing for the calculation of cumulative risk during the hazard period based on an established annual risk. In the example, the CRR denominator consists of the risk of HIE in a neonate exposed to chorioamnionitis, abnormal fetal heart rate, and maternal obesity, in the absence of placental abruption. Epidemiologic data shows this value to be 1.7% (1 in 59).
- Calculation of the CRR: the numerator is divided by the denominator to arrive at the CRR. In this case, the CRR is thus:
  $$\mathrm{CRR}\;=\;\frac{14.9}{1.7}=8.76$$
- The CRR can be converted into a probability of causation (PC) [15], as follows:
  $$\mathrm{PC}=\frac{\left(\mathrm{CRR}-1\right)}{CRR}\times 100\%=\frac{\left(8.76-1\right)}{8.76}\times 100\%=88.58\%$$

This means that there is an 88.58% probability that in this case the neonate’s HIE was caused by the placental abruption in addition to all the other risk factors known to be present. The PC can then be compared to the required standard of proof set for the expert witness evidence as a part of the whole evidence being considered in the legal venue.

**Figure 2:**
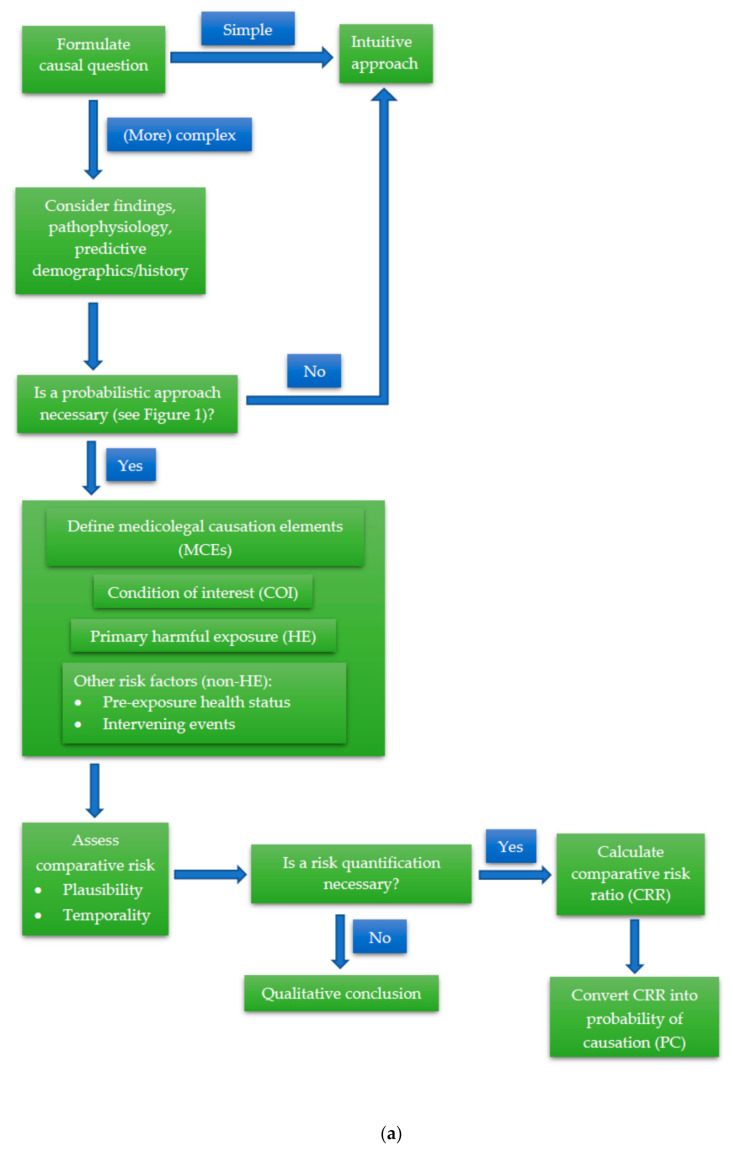

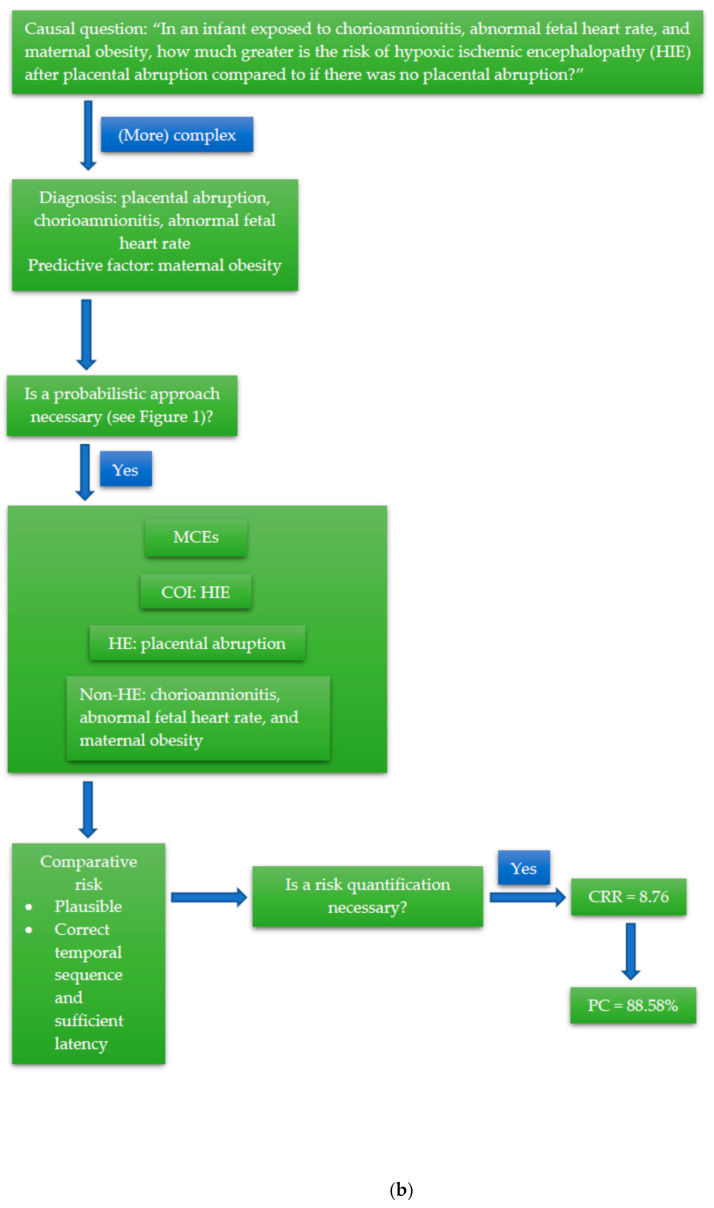
(**a**) Schematic of the INtegration of Forensic Epidemiology and the Rigorous EvaluatioN of Causation Elements (INFERENCE) approach. (**b**) Example of the INFERENCE approach in causal analysis of a case of fetal hypoxic ischemic encephalopathy (HIE).

Figure 2a below depicts the whole process of INFERENCE as a flowchart or decision tree and Figure 2b shows how the INFERENCE approach is used in the HIE example.

## 4. Discussion

We have developed a causal analysis approach that is based on counterfactual inference and produces a PC to be compared to the relevant standard of proof. Any method or approach used in causal inference should provide simple guidance, perform adequately, and be scientifically sound (“academic”), and yet still be practical and simple enough to be used in daily practice [6,32,33]. The INFERENCE approach may not be suitable or necessary in many cases. The diagram shown by Figure 1 can help in the determination of when the approach is most appropriate. In general, the higher the number of competing causes, the heavier the reliance on Hill’s viewpoints, and the greater the need for a CRR, the more we could benefit from the INFERENCE approach. On the other hand, if the causal relationship “makes sense” based on scientific common-sense, medical heuristics, categorical intuitive deduction, and professional experience, then the intuitive approach may suffice. In cases where the causal relationship does not immediately “make sense”, but the relationship is not too complicated, a hybrid approach can be used as a middling. This hybrid approach uses a stepwise and logical thinking methodology but is not as laborious as the INFERENCE approach and might not produce a quantifiable probability of causation.

There are several strengths of the INFERENCE approach. It provides a systematic methodology for medicolegal causal analysis, which can help ensure that all potential causation elements are assessed in an orderly manner, and no elements are overlooked. Additionally, as the INFERENCE approach becomes part of standard evidence-based practices, it can be used to assess expert performance, especially in terms of “within-expert” and “between-expert” reliability in drawing conclusions [34]. In other words, if experts use the same approach, they will most probably get to the same conclusion; hence, improving the reproducibility of expert opinions. Furthermore, using a standard approach to arrive at a conclusion improves the transparency of an expert opinion. This improvement in transparency is achieved by making the cognitive process of the expert accessible to the reader, i.e., the reader can follow the thinking process of the expert step by step. As the approach uses (forensic) epidemiologic principles, methods, and data, it can also be suited to the specific population demographics and characteristics of the victim/plaintiff. Hence, the obtained CRR (and PC) are better reflections of the circumstances of the case at hand, and not just theoretical. The INFERENCE approach can also utilize and incorporate a variety of epidemiologic study designs and strategies to obtain the necessary data through ad hoc studies if the data are not readily available (or accessible) from existing databases and literature. These additional methodologies encompass simple cross-sectional studies up to clinical trials and even outbreak/cluster investigations.

The approach is also more objective than the intuitive approach, which is the current practice in forensic medicine, because it produces conditional probability based on a given set of known circumstances. It is also compatible with the requirements of legal proceedings because it produces a quantitative result (in the form of a PC) that reflects the level of certainty and that can be directly compared to the required standard of proof. As an additional benefit, the approach is inherently transparent and nonpartisan and thus can be used by both parties involved in a legal proceeding.

There are some potential drawbacks of the INFERENCE approach. The approach is based on Bayesian principles, in that it applies probabilistic reasoning to factual scenarios to arrive at the ultimate conclusion. As such, as with nearly all causal assessments, the results of an analysis using the INFERENCE approach are highly dependent on the relevant information available at the time of analysis. Subjectivity is also introduced into the analysis when evaluating all plausible nontrivial competing causes, as the assessment may vary given the knowledge and experience of the forensic medical practitioner. Additionally, the INFERENCE approach requires more from the forensic medical practitioner regarding time, effort, and specialized expertise, including an understanding of basic principles of epidemiology and medical statistics, along with the other specialized medical knowledge needed for the analysis. Finally, in using INFERENCE, it is important to remember that any causal analysis is inherently retrospective, and that it is impossible to be 100% certain that the result is indeed the truth. Causal analyses are also always complex, in that they require a learned thinking process, even when completed using professional intuition (i.e., intuition that is obtained through years of professional education, training, and experience). Thus, rather than just relying on the end result, i.e., the CRR/PC obtained from the formula, as a “key” to the answer, the INFERENCE approach should be scrutinized as one whole process in which each step builds on the previous one towards a justified probabilistic opinion.

## 5. Conclusions

We present an evidence-based approach for the assessment of specific causation in forensic medicine using forensic epidemiological principles, called “The INFERENCE (INtegration of Forensic Epidemiology and the Rigorous EvaluatioN of Causation Elements) Approach.” We have also described a means of identifying the circumstances in which the approach is most beneficial. INFERENCE is intended to be used as a real-world approach to evidence-based causal analysis. The goal of the INFERENCE causal approach is to improve common practice standards despite a diversity of local specific practices, thereby promoting quality assurance in forensic medicine and providing more reliable expert opinions in legal proceedings.

## Figures and Tables

**Table 1:** Elements of causal analysis approaches.

Category of Causal Analysis Methods	Intuitive	Probabilistic	
		Hybrid	INFERENCE
Formulation of a causal question	(+)	(+)	(+)
Consideration of examination findings, injury/pathophysiologic mechanism, and predictive demographics and history	(±)	(+)	(+)
Definition of the medicolegal causation elements (MCE)		(+)	(+)
Comparative risk assessment of competing causes (plausibility, temporality)		(+)	(+)
Quantification of comparative risk			(+)
Case type examples	High risk, single suspect cause, e.g.,: Gunshot wound to the head/decapitation injury	Multiple suspect cause (requiring comparison of risk), but without need to quantify risks precisely, e.g.,: Risk of intracranial bleeding due to suspected (unproven) shaken-baby syndrome versus spontaneous bleeding due to known factor VII deficiency	Multiple suspect causes and the need for quantitative comparisons via comparative risk ratio (CRR) analysis, e.g.,: Increase in risk of hypoxic-ischemic encephalopathy due to placental abruption versus due to other known risk factors without placental abruption

## Appendix A

**Table A1:** Current approaches to medicolegal causal analysis [9].

No.	Approach	Category	Application	Example	Strengths	Weaknesses
**1.**	Intuitive approach (i.e., scientific common-sense)	Intuitive	Simple cases where the causal relationship “makes sense” based on fundamental scientific principles	Death following a gunshot wound to the head	Practical, does not need exceptional/additional resources	Not suitable for more complex cases where the causal relationship is not as readily apparent
**2.**	Categorical intuitive deduction (i.e., the Sherlock Holmes style or educated guess)	Intuitive	Cases where there is only one plausible cause at the same time	Death following ingestion of insecticides in a previously healthy person with no signs of trauma	Impressive expert witness testimony	Not suitable for more complex cases where there is more than one plausible cause, requires a lot of professional experience, potentially misleading
**3.**	Hill’s viewpoints	Intuitive-probabilistic	Cases with sufficient epidemiologic evidence and literature to assess competing causal hypotheses	Post-traumatic headache in a sexual-assault victim	Check-list-like criteria to guide causal inference	Temporal sequence is the only real “causal criterion”, the meaning or value of the other criteria can be unclear
**4.**	The American Medical Association (AMA) Guides to the Evaluation of Disease and Injury Causation	Intuitive-probabilistic	Primarily cases of injury with multiple plausible causes and work-related conditions	Lower back-pain in a factory worker who stands all-day	Provide elements that may be used for a systematic step-by-step causal analysis, primarily to assess work-relatedness	Lengthy and complicated process, does not produce a PC/quantification of the level of certainty
**5.**	Forcier-Lacerte medicolegal causal analysis model	Intuitive-probabilistic	Primarily cases of injury with multiple plausible causes and cases related to insurance claims	An elderly woman with severe osteoporosis who sustains a slip-and-fall resulting in several fractured ribs	Provide elements that may be used for a systematic step-by-step causal analysis, categorizes possible causes into (1) the accident, (2) preexisting health status, and (3) intervening event.	Lengthy and complicated process, does not produce a PC/quantification of the level of certainty
**6.**	The epidemiology-based approach by Siegerink et al.	Probabilistic	Civil litigations or cases of tort, where the issue is primarily about the proportional liability of multiple plausible causes	The risk of lung cancer in a factory worker exposed to asbestos, who is also a heavy smoker with a family history of lung cancer	Fits both the sufficient cause model and the counterfactual model, offers proportional liability for each component cause (i.e., the unlawful act plus other possible factors)	Could overestimate the number of components of the sufficient cause, leading to an underestimation of liability, all components are considered as of equal importance, while from a legal perspective some causes may be more important than others (e.g., unlawful act vs genetics)
**7.**	The 3-step medicolegal causation approach by Freeman	Probabilistic	Cases of injuries with multiple plausible causes that do not require a high degree of energy, preexisting conditions symptomatic after relatively minor trauma, or conditions with an insidious symptom onset	An elderly woman with shoulder pain after a minimal-damage rear-impact collision	Practicable, systematic, fits the standards of both medical and legal practice by establishing (1) plausibility, (2) temporality, and (3) the absence of a more probable alternative explanation (differential etiology)	Requires sufficient epidemiologic data and comprehension of epidemiologic methods to compare risks of differential etiologies
**8.**	The forensic epidemiology approach	Probabilistic	Highly complex cases with multiple plausible causes	Peripartum cardiomyopathy in a young woman following exposure to doxorubicin	Systematic, provides a scientifically valid and verifiable quantification of probability in the form of a comparative risk ratio (CRR) and a probability of causation (PC), results are suitable for presentation in a court of law	Uses epidemiologic principles, methods, and data to formulate a probability, the analyses and calculations can be quite complicated, might not be suitable for day-to-day forensic medical practice

## Appendix B

**Table A2:** Overview of Hill’s viewpoints.

Viewpoint	Definition [18,35]	Forensic Example
Strength of association	A strong association, which is typically indicated by a high relative risk (RR), is more probably to be causal.	The association between a GSW to the head and death is generally speaking very strong because the risk of dying from a GSW to the head is extremely high. On the other hand, not all patients with septic shock die, and neither do all patients receiving opiate injections. Thus, the risk of dying from either cause is not as high as from a GSW to the head.
Consistency	If an association is observed in different settings or circumstances, it is probably causal.	That a person who has been shot in the head dies because of it has been observed repeatedly in various settings. Meanwhile, the death of a patient with septic shock is subject to a variation of circumstances, with or without opiate injections.
Specificity	If an effect is significantly associated with a certain cause or *vice versa*, then their relationship is probably causal.	A GSW to the head very specifically leads to death (although miraculous recoveries do occur very rarely). The relationship between septic shock and death, and between opiate injections and death, is, however, not as specific.
Temporality	The suspected cause must precede its effect, with a (biologically) appropriate sufficiency and latency (window period).	In cases of deaths, the temporal sequence is rarely an issue. What must be considered are two other aspects of temporality, i.e., temporal plausibility and temporal latency. In a GSW to the head, death often occurs instantaneously. Therefore, the causal relationship is usually plausible timewise, and the existence of competing causes occurring in the temporal window between the GSW and death (e.g., myocardial infarction) is highly unlikely. In contrast, a patient with septic shock dying, say, 6 h after injection of a short-acting opiate is less plausibly related to the injection than if death occurred in 30 min. Additionally, more competing causes could have occurred in the temporal window, which must be considered.
(Biological) plausibility	The degree to which the association is explainable by (currently known) scientific principles.	That a GSW to the head is (almost certainly) incompatible with life is consistent with known biological and scientific principles. The relationship between septic shock, opiate injections, and death is, however, subject to closer scrutiny as there are various biologic factors that could influence the outcome.
Coherence	The causal relationship may not seriously conflict present fundamental scientific facts.	In the simplest of words, it “makes sense” that a person who has been shot to the head subsequently dies. On the other hand, we would possibly be more surprised to find a patient dying after receiving an opiate injection that has been given appropriately.
Analogy	Known causal relationships between similar causes and effects may be translatable to unknown causal investigations.	A GSW to the head using a shotgun and a small-caliber bullet can be considered analogous in that it usually results in the same outcome, i.e., death. Thus, the exposure is translatable from one GSW case to the other. In the opiate-case, however, what can be considered as analogous is harder to define. More factors (e.g., from the patient, the injection, even the person giving the injection) need to be comparable be able to translate the exposure between cases.
Experiment	In some cases, experimental studies can provide evidence of causality.	It might be quite impossible to find experimental evidence for the GSW to the head (as with many exposures to trauma in forensic medical cases). For the opiate injection, however, randomized-controlled experiments are available and should be considered.
Dose-response relationship	A bigger “dose” (e.g., greater exposure) usually leads to a greater effect.	This viewpoint is more appropriate in the opiate injection case as the relationship between the dose of the opiate preparation and death is directly proportional. Nevertheless, it could also apply to the GSW if we consider the bullet caliber, shooting distance, etc. as the “dose”.

All examples in this table use a scenario of death following a gunshot wound (GSW) to the head versus the death of a patient with septic shock after an opiate injection.
